# Mimicking the Biology of Engineered Protein and mRNA Nanoparticle Delivery Using a Versatile Microfluidic Platform

**DOI:** 10.3390/pharmaceutics13111944

**Published:** 2021-11-17

**Authors:** Valentina Palacio-Castañeda, Rik Oude Egberink, Arbaaz Sait, Lea Andrée, Benedetta Maria Sala, Negar Hassani Besheli, Egbert Oosterwijk, Johan Nilvebrant, Sander C. G. Leeuwenburgh, Roland Brock, Wouter P. R. Verdurmen

**Affiliations:** 1 Department of Biochemistry, Radboud Institute for Molecular Life Sciences, Radboud University Medical Center, Geert Grooteplein 28, 6525 GA Nijmegen, The Netherlands; valentina.palacio-castaneda@radboudumc.nl (V.P.-C.); rik.oudeegberink@radboudumc.nl (R.O.E.); arbaaz.arbaaz@student.ru.nl (A.S.); 2 Department of Dentistry—Regenerative Biomaterials, Radboud Institute for Molecular Life Sciences, Radboud University Medical Center, Philips van Leydenlaan 25, 6525 EX Nijmegen, The Netherlands; lea.andree@radboudumc.nl (L.A.); Negar.HassaniBesheli@radboudumc.nl (N.H.B.); sander.leeuwenburgh@radboudumc.nl (S.C.G.L.); 3 Division of Protein Engineering, Department of Protein Science, School of Engineering Sciences in Chemistry, Biotechnology and Health, AlbaNova University Center, Royal Institute of Technology, SE-100 44 Stockholm, Sweden; bmsala@kth.se (B.M.S.); johanni@kth.se (J.N.); 4 Department of Urology, Radboud Institute for Molecular Life Sciences, Radboud University Medical Center, Geert Grooteplein 26/28, 6525 GA Nijmegen, The Netherlands; egbert.oosterwijk@radboudumc.nl; 5 Department of Medical Biochemistry, College of Medicine and Medical Sciences, Arabian Gulf University, Manama 329, Bahrain

**Keywords:** drug delivery, affibody, CAIX, gelatin, mRNA, nanoparticle, microfluidics, nanomedicine, biomaterial

## Abstract

To investigate the delivery of next-generation macromolecular drugs, such as engineered proteins and mRNA-containing nanoparticles, there is an increasing push towards the use of physiologically relevant disease models that incorporate human cells and do not face ethical dilemmas associated with animal use. Here, we illustrate the versatility and ease of use of a microfluidic platform for studying drug delivery using high-resolution microscopy in 3D. Using this microfluidic platform, we successfully demonstrate the specific targeting of carbonic anhydrase IX (CAIX) on cells overexpressing the protein in a tumor-mimicking chip system using affibodies, with CAIX-negative cells and non-binding affibodies as controls. Furthermore, we demonstrate this system’s feasibility for testing mRNA-containing biomaterials designed to regenerate bone defects. To this end, peptide- and lipid-based mRNA formulations were successfully mixed with colloidal gelatin in microfluidic devices, while translational activity was studied by the expression of a green fluorescent protein. This microfluidic platform enables the testing of mRNA delivery from colloidal biomaterials of relatively high densities, which represents a first important step towards a bone-on-a-chip platform. Collectively, by illustrating the ease of adaptation of our microfluidic platform towards use in distinct applications, we show that our microfluidic chip represents a powerful and flexible way to investigate drug delivery in 3D disease-mimicking culture systems that recapitulate key parameters associated with in vivo drug application.

## 1. Introduction

While drug development efforts have traditionally focused on small-molecule drugs, during the last decade, there has been an increasing emphasis on biologics as novel therapeutic agents [1], which include various types of proteins, notably antibodies, oligonucleotides, viral gene therapies, and nanoparticle-based drug formulations. Since these biologics are large compared to small molecules, they cannot cross cellular membranes simply by diffusion. Additionally, transport through tissue may be hampered, thereby complicating the accessibility of biologics to target molecules or subcellular localizations required for activity.

While animal models have classically been utilized to study drug delivery challenges in 3D environments, these models face several drawbacks when utilized for the investigation of next-generation targeted biological therapies. Importantly, due to a highly exquisite binding specificity, targeting agents such as binding proteins are often fully or partially species-specific, thus negatively affecting extrapolation of results from animal to human in some applications [2,3]. This challenge extends to intracellular targets or intracellular effects of proteins with therapeutic activities that are expressed in the cytosol, e.g., by mRNA-based therapies. Additional challenges are low throughput, the inability to properly control for key parameters in animal experiments such as timing, dose, and monitoring of distribution, and ethical issues that are associated with the use of animals in scientific research, in particular if the animal models only partially recapitulate the human situation.

Because of these drawbacks, the utilization of 3D cell-culture approaches and organ-on-a-chip systems has recently gained considerable interest. While 3D cell-culture systems, such as organoids, recapitulate the 3D architecture of (human) tissues [4,5], they often fail to incorporate essential biophysical and biochemical cues, such as flow, pressure, controlled oxygen gradients, and proper spatial compartmentalization. Hence, organ-on-a-chip systems have come to the foreground as a complementary approach to study disease and effects of therapeutic agents; in particular, to study transport phenomena [4,6,7]. In our research, we have recently implemented organ-on-a-chip technology for the investigation of drug delivery in various contexts and reported novel insights for retargeted adenoviral vectors and therapeutic proteins targeting various cell surface receptors overexpressed on tumor tissue [8,9]. Notably, we were able to link experimental observations to predictions made by mathematical modeling of drug delivery, ultimately guiding a rational design of protein therapies [10]. 

Hence, our microfluidic system can be used to study drug delivery characteristics of promising therapeutic proteins at high resolution. Carbonic anhydrase IX (CAIX) is a surface receptor that is widely overexpressed in hypoxic regions of tumors as its expression is upregulated upon stabilization of hypoxia-inducible factor 1α (HIF-1 α) under hypoxic conditions [11]. The protein internalizes rapidly and therefore serves as an excellent target for drug delivery to hypoxic regions [12] or to renal tumors, where hypoxia-signaling processes are aberrantly activated due to mutations in von Hippel Lindau (VHL) [13]. Affibodies are a group of non-antibody binding proteins, and variants engineered for specific targeting of CAIX have been previously developed and reported to target CAIX in vitro as well as in vivo [14]. 

Microfluidic systems also provide advantages for the design of biomaterials as carriers for local drug delivery. Biomaterials are intended for application in the human body, which means that they need to function in a highly complex biological environment. In order to faithfully recapitulate interactions of cells with the biomaterial, cellular growth characteristics should mimic physiological conditions in tissues. From a practical perspective, the miniaturized geometry of a microfluidic system minimizes sample consumption during the development phase, which is a relevant asset in the development of costly therapeutic biomaterials and therapeutics such as mRNA or other types of oligonucleotides.

Here, we build upon our work on 3D culture systems and organ-on-a-chip technology for the investigation of drug delivery. We illustrate the affibody-mediated targeting of CAIX on-chip, as well as the nanoparticle-mediated delivery of mRNA expressing the reporter protein eGFP from colloidal gelatin gels in the context of biomaterial development for bone regeneration. Our results demonstrate the versatility of our microfluidic platform for a microscopy-based investigation of the biology of drug delivery of engineered proteins and nanoparticles in highly tunable, in vivo-mimicking conditions. 

## 2. Materials and Methods

### 2.1. Cell Lines and Culture Media

The CAIX targeting experiments were performed with human renal carcinoma cells classified as CAIX-positive (SK-RC-52) or CAIX-negative (SK-RC-17) [15]. Both cell lines were cultured in Roswell Park Memorial Institute medium (RPMI), supplemented with 10% fetal bovine serum (FBS; Gibco, Waltham, MA, USA, Catalog number: 10270-106) and 1× Glutamax (Life Technologies, Carlsbad, CA, USA). Subconfluent cell cultures of the MC3T3-E1 subclone 4 (CRL-2593, American Type Culture Collection, Manassas, VA, USA) preosteoblastic murine cell line were maintained in Minimal Essential Medium α (MEM-α; Gibco, Catalog number: A10490-01) supplemented with 10% FBS. For the experiments with microfluidic devices, 100 units mL^−1^ penicillin and 0.1 mg mL^−1^ streptomycin (Cat. No. P0781; Sigma-Aldrich, St. Louis, MO, USA) and 2.5 µg mL^−1^ Amphotericin B (Sigma-Aldrich) were added to the media.

### 2.2. Cloning, Expression, and Purification of Affibodies

The CAIX-binding affibody (ZCAIX-2) and the Taq polymerase-binding affibody ZTAQ, serving as a non-binding control affibody in this study, were reported previously [14,16]. The affibodies were cloned with an N-terminal His_6_ tag and a C-terminal mCherry tag in a T7 expression plasmid. The mCherry-fused affibodies were expressed in the E. coli strain BL21(DE3) and purified essentially as described previously [17], with the exception that 2YT medium containing 50 μg mL^−1^ kanamycin (Life Technologies, Carlsbad, CA, USA) was used for the protein expression. 

### 2.3. Microfluidic Chip Manufacturing and Functionalization 

**Figure 1:**
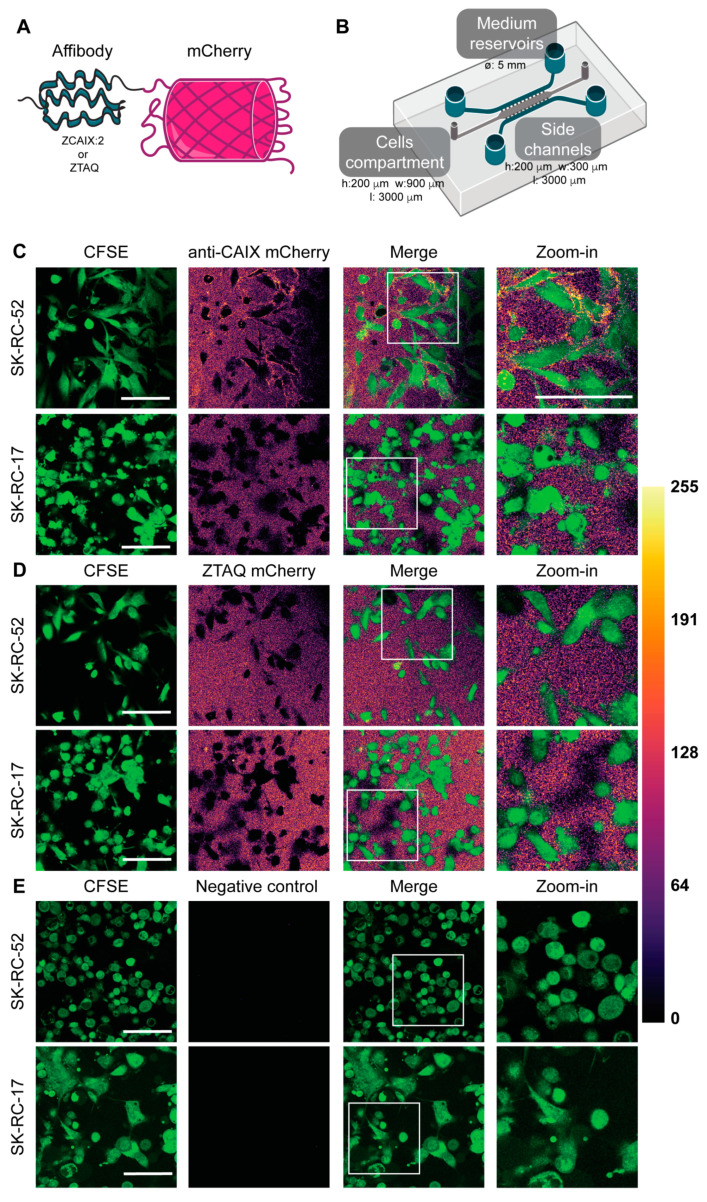
Affibody-mediated CAIX targeting on-chip. (**A**) Schematic representation of an affibody fused to mCherry. (**B**) Schematic representation of the microfluidic chip. (**C**,**D**) Confocal microscopy images of a tumor-on-a-chip after a one-hour incubation of CAIX-positive SK-RC-52 (**C**) or CAIX-negative SKRC-17 cells (**D**) with the anti-CAIX affibody-mCherry fusion or the ZTAQ affibody-mCherry fusion, which was used as non-binding control. (**E**) Medium only was used as a negative control to determine background fluorescence. The mpl-inferno LUT was used to depict the intensity in the mCherry channel. Scale bars represent 100 µm. CFSE, carboxyfluorescein succinimidyl ester.

A previously published design of the microfluidic chip was used [9]. Briefly, the device, consisting of three parallel channels connected by narrow microchannels (for chip dimensions, see Figure 1B), was fabricated utilizing replica-molded polydimethylsiloxane (PDMS; Dow Corning, Midland, MI, USA) and bonded to a 1 mm thick, 75 × 25 mm plain glass microscopy coverslip or to SuperFrost Plus glass slides with 75 × 25 mm and 175 ± 5 µm thickness (Menzel Gläser, Braunschweig, Germany). Bonding was performed with a Basic Plasma Cleaner (230 V, Harrick Plasma, Ithaca, NY, USA). Reservoirs connected to the side channels were made by punching 5 mm holes into the PDMS using a biopsy puncher (Kai Industries, Seki, Gifu, Japan). Before cell seeding in the chips, the devices were functionalized with a solution of 2 mg mL^−1^ dopamine hydrochloride in 10 mM Tris-HCl, pH 8.5, to improve collagen or gelatin attachment to the PDMS by enhancing surface wettability of the PDMS [18].

### 2.4. CAIX Targeting in Microfluidic Tumors-on-Chips and Confocal Laser Scanning Microscopy (CLSM)

CAIX-positive SK-RC-52 cells and CAIX-negative SK-RC-17 cells were labeled with carboxyfluorescein succinimidyl ester (CFSE, ThermoFisher Scientific, Waltham, MA, USA) according to the manufacturer’s instructions. The labeled cells were then resuspended in a 4 mg mL^−1^ rat tail collagen type I (Corning, Corning, NY, USA) gel and loaded manually in the tumor compartment of the microfluidic chip at a density of 1.5 × 10^7^ cells mL^−1^. The chips were placed in the incubator at 37 °C for 15 min to allow the collagen to polymerize. After this time, complete medium was added in the side channels, each of the four reservoirs was filled with complete medium, and the top of the chips was covered with complete medium to prevent drying of the collagen. The chips were placed in a Petri dish covered with a lid to reduce evaporation. After 24 h, affibody-mCherry diffusion through the collagen matrix and the specific targeting of CAIX-positive SK-RC-52 cells or control cells was investigated by adding anti-CAIX-mCherry affibody or the non-binding ZTAQ-mCherry affibody at a concentration of 500 nM in both side channels and reservoirs. Cellular targeting of the affibody fusions was imaged after 1 h using a Leica TCS SP8 confocal microscope (Leica Microsystems, Mannheim, Germany) equipped with an HCX APO 40×/0.75 dry objective (Leica Microsystems) and a white-light laser. Images were acquired of at least three positions in the chips in two independent experiments. Z-stacks were also collected in each chip. Fiji was used for image processing [19]. CFSE was excited at 492 nm (detection: 500–520 nm), and the mCherry-fused affibodies were excited at 550 nm (detection: 558–650 nm).

### 2.5. Messenger RNA (mRNA)

Enhanced green fluorescent protein (eGFP) mRNA (L-7601) and 5-methoxyuridine-substituted Cyanine 5 (Cy5)-labeled eGFP mRNA (L-7701) were purchased from Trilink Biotechnologies (San Diego, CA, USA). Both mRNAs had a length of 996 nucleotides (nt), were capped using CleanCap technology, and polyadenylated (276 nt). Cy5-eGFP mRNA contained a 5-UTP:5-Methoxy-UTP substitution at a ratio of 1:3; the translation efficiency is known to correlate inversely with Cy5-UTP substitution [20]. All mRNA was snap-frozen in liquid nitrogen and stored at 100 ng µL^−1^ in Milli-Q (MQ) in DNA LoBind tubes (Eppendorf, Hamburg, Germany) at −80 °C until use. Before use, the mRNA solution was thawed and kept on ice.

### 2.6. Transfection Complex Formation

Cell-penetrating peptide PepFect14 (PF14) with the following sequence: Stearyl-AGYLLGKLLOOLAAAALOOLL-NH_2_, where O denotes the non-proteinogenic amino acid ornithine and -NH_2_ indicates a C-terminal amidation, was obtained from EMC Microcollections (Tübingen, Germany). The peptide was dissolved in MQ at a concentration of 0.75 mM in Protein LoBind tubes and incubated at room temperature (RT) for 20 min under gentle agitation before aliquots were snap-frozen in liquid nitrogen and stored at −20 °C. 

Before the formation of transfection complexes, benchtops, glass, plasticware and pipettes were thoroughly cleaned with RNAse AWAY Surface Decontaminant (ThermoFisher). Polycationic peptide-based complexes (polyplexes) were formed by a ‘50/50 stream method’. Two separate stock solutions of mRNA and PF14 were prepared in MQ and simultaneously aspirated with electronically dispensing pipettes (E4 Electronic Pipette, LTS E4-100XLS+, Mettler-Toledo Rainin, LLC, Oakland, CA, USA) at a flow rate of 11 mL min^−1^. The pipette tips were inserted into a custom-made 1.5 mL Eppendorf tube holder to collect the solution. The angle between both pipette tips was 75°, and the angle between the tips and the tube wall was 45°. PF14 polyplexes were formed at a concentration of 40 µM (ten times concentrated) at a nitrogen/phosphate (N/P) ratio of 3, which corresponds to 80.4 nM mRNA and a molar ratio of 497.5 PF14:mRNA.

For the formation of polycationic lipid-based complexes (lipoplexes), Lipofectamine MessengerMAX (LMM; ThemoFisher Scientific) was used as per the manufacturer’s instructions. In short, LMM was incubated in a volume of Opti-MEM (Gibco, Cat. No. 11058021) at a ratio of 1:32.34 for 10 min at RT. mRNA solution was diluted in Opti-MEM at a ratio of 1:4 and incubated with LMM for at least 5 min at RT. This yielded a lipoplex mixture with a concentration of 10 ng mRNA µL^−1^.

For quality control, the hydrodynamic size of the nanoparticles was measured at 25 °C by dynamic light scattering (DLS) using a Zetasizer Nano ZS (Malvern Instruments, Worcestershire, UK) equipped with a 4 mW He-Ne laser (633 nm) with a backscatter detection angle of 173°. A total 40 µL of ten-times-concentrated NP (both PF14 and LMM) solution was measured in a UV-cuvette (BrandTech Scientific, Essex, CT, USA, Cat. No. 759200).

### 2.7. Synthesis of Colloidal Gelatin Nanoparticles

Positively charged gelatin A (Gel A; Bloom number 285) and negatively charged gelatin B (Gel B; Bloom number 247) powders were kindly provided by Rousselot (Rousselot, Ghent, Belgium). Gelatin NPs were made using an acetone-based desolvation method, as previously described [21]. In brief, gelatin powder was dissolved in demi-water at 5% *w/v* while stirring at 400 rpm at 40 °C. After dissolution, the pH was adjusted to 2.5 with 6 M HCl (37% *w*/*v* fuming, Merck Millipore, Burlington, MA, USA). Thereafter, 135 mL of acetone (Boom, Meppel, The Netherlands) was added at a flow rate of 8 mL min^−1^ while vigorously stirring (1000 rpm), which induces desolvation of gelatin into spherical NPs. After cooling down, the NPs were crosslinked with 316 µL of 25% glutaraldehyde (Acros Organics, Geel, Belgium) and stirred at 400 rpm overnight at RT. The next day, 100 mL of 100 mM glycine solution (Sigma-Aldrich) was added to capture unreacted glutaraldehyde. NPs were collected by centrifugation (40 min at 16,800× *g*, 25 °C) and washed twice with demi-water. Afterward, the washed NPs were redispersed in a 30/70% *v/v* mixture of acetone and demi-water, snap-frozen in liquid nitrogen, and lyophilized for 48 h.

### 2.8. mRNA Transfection in Microfluidic Chips and CLSM

One day before transfection, 1 × 10^5^ MC3T3-E1 cells, in a volume of 20 µL, were seeded in the main channel of the microfluidic chip, and cells were allowed to adhere to the glass slide for 4 h. Once adherent, the reservoirs were filled with 200 µL of complete MEM α. 

The next day, PF14 polyplexes with Cy5-eGFP mRNA were pre-diluted to a peptide concentration of 8 µM, mixed with an equal volume of 4% *w*/*v* colloidal gelatin NPs, both in complete MEM α, of which 20 µL was immediately added to the main channel of the microfluidic chip. The mRNA input amounted to 51.5 ng per chip (8.04 nM mRNA). Importantly, LMM lipoplexes were diluted such that the mRNA inputs were equal for both types of transfection complexes. Two hours post-transfection, cellular uptake of the transfection complexes was visualized using the Leica TCS SP8 SMD (Leica Microsystems) equipped with an HCX PL APO 10×/0.40 dry objective and a temperature-controlled stage at 36.5 °C. Cy5 was excited with a white-light laser at 633 nm, and emission was collected between 650 and 690 nm using a photomultiplier tube (PMT) detector. To visualize the entire microfluidic chip, tile scanning was performed with 10% overlap, followed by stitching with the mosaic merge function in Leica Application Suite X software (version 3.7.2.22383).

For enhanced resolution, eGFP expression was assessed 24 h post-transfection using an LSM 900 (Carl Zeiss, Oberkochen, Germany) equipped with a W Plan Apochromat 40×/1.0 DIC VIS-IR M27 objective. eGFP was visualized by excitation with an argon-ion laser at 488 nm, and fluorescence was collected between 500 and 550 nm using a GaAsP-PMT. To visualize the entire microfluidic chip, tile scanning was performed with 10% overlap, followed by stitching.

## 3. Results

### 3.1. CAIX Targeting in Microfluidic Tumors-on-Chips 

To investigate the targeting of CAIX in a 3D microfluidic tumor-on-a-chip, we first generated affibodies fused to mCherry. A previously reported CAIX-targeting affibody (ZCAIX-2) was used for CAIX targeting, whereas the Taq polymerase-binding affibody ZTAQ served as a negative control [14,16]. Proteins were produced in E. coli, and a one-step purification yielded pure proteins (Figure S1A). We first confirmed their targeting specificity in 2D on CAIX-positive SK-RC-52 cells and CAIX-negative SK-RC-17 cells (Figure S1B). The anti-CAIX affibody yielded a clear membrane staining in the CAIX-positive cells, which was absent in the negative cells. No staining was observed for the control ZTAQ affibody.

After confirmation of exclusive binding to CAIX-overexpressing cells in 2D, the targeting of CAIX by the affibodies was tested in 3D in our microfluidic tumor-on-a-chip. The morphology of cells cultured in 2D (Figure S1B) differs greatly from the morphology of cells that grow embedded in a collagen matrix (Figure 1 and Figure S1C). We furthermore noted differences between different areas of the microfluidic chip (Figure S1C). Cells that grow attached to the glass have a stretched morphology similar to 2D cell cultures on plastic, whereas cells embedded in the collagen matrix present a more rounded phenotype. Through compartmentalization inherent in the design, we were able to add the affibody solutions to the blood vessel-mimicking side channels. By diffusion through narrow channels connecting the side and middle compartments, which mimics the process of extravasation through a leaky endothelium [9], we were able to subsequently image the enrichment at the plasma membranes of cells incorporated as a solid 3D tumor mass after diffusion through the collagen matrix in the middle compartment. 

At the 24 h timepoint after loading the tumor cell mass, mCherry-fused anti-CAIX or non-binding ZTAQ affibodies were added to the chips containing either CAIX-positive SK-RC-52 or CAIX-negative SK-RC-17 cells. Cells were imaged after one hour using confocal microscopy. Based on previous work in which we showed targeted protein delivery using another non-antibody protein scaffold in a similar configuration of the microfluidic device, we selected 1 h as a fixed timepoint. After 1 h, we expected that the affibodies would have diffused through the matrix and reached all cells [9]. We observed a clear membrane staining on the surface of the SK-RC-52 CAIX-positive tumor cells throughout the collagen matrix and no binding to the membrane on CAIX-negative SK-RC-17 cells (Figure 1C and Supplementary Videos S1 and S2). As expected, the non-binding ZTAQ affibodies did not show such enrichment on the membrane of either cell line (Figure 1D and Supplementary Videos S3 and S4). When employing similar settings as for mCherry signal detection, there was no detectable signal for the medium, cells, or collagen matrix (Figure 1E). These results confirm the ability of the anti-CAIX affibody-mCherry fusion to target CAIX in a complex 3D environment and represent the first example of targeting CAIX in a microfluidic tumor-on-a-chip. Relative to the 3D cell culture, the lower background level in 2D cell cultures is due to the additional wash steps in 2D. Imaging in the 3D culture was done without washing away the proteins.

### 3.2. Delivery of mRNA from Colloidal Gelatin within a Microfluidic Chip

To assess the compatibility of positively (Gel A) or negatively charged (Gel B) colloidal gelatin nanoparticles with formulations for mRNA transfection, peptide- (PF14) and lipid-based mRNA complexes (LMM) were formed and characterized by DLS (Figure S2). PF14 nanoparticles (NPs) had an average size of 80.2 ± 1.9 nm (Figure S2A), while LMM NPs had an average size of 525.8 ± 105.2 nm (Figure S2B). Furthermore, the PF14 NPs showed a monodisperse particle-size distribution, while LMM NPs displayed multiple peaks of different sizes. On average, the diameter of PF14 NPs was 6.3 times smaller than that of LMM-based mRNA NPs (Table S1).

**Figure 2:**
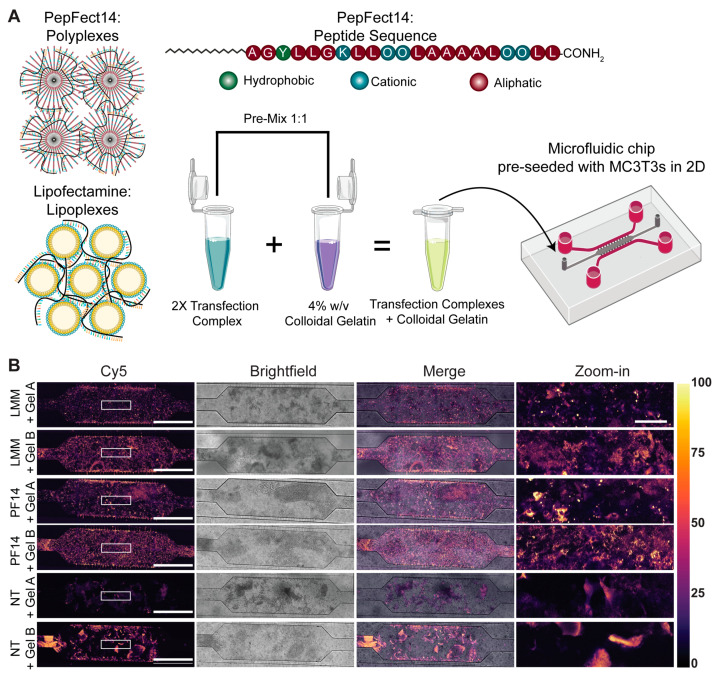
Uptake of mRNA transfection complexes in the presence of colloidal gelatin. (**A**) Schematic overview of mRNA transfections in the presence of colloidal gelatin within a microfluidic chip. PF14 forms micelles due to its stearyl moiety, and due to its positive charge, it electrostatically complexes mRNA into polyplexes. LMM lipoplexes are pre-formed lipid micelles that complex mRNA via their cationic lipid head groups. Image not drawn to scale. (**B**) Confocal microscopy images of uptake into MC3T3 cells two hours post-transfection, where the Cy5-label of Cy5-eGFP mRNA is visualized. For enhanced clarity, an enlarged region of interest, which corresponds to the white rectangle (width: 736 µm, height: 209 µm) in the leftmost column, is shown in the rightmost column. The untreated conditions were used as negative controls to assess the autofluorescence of colloidal gelatin. The mpl-inferno LUT depicts the Cy5 intensity, and all Cy5 images were calibrated equally across conditions. Scale bars represent 1000 µm; scale bars in zoom-in panels represent 150 µm. Cy5, Cyanine5; NT, non-treated.

Following the successful formation of mRNA transfection complexes, the cellular uptake of these NPs by preosteoblastic cells upon delivery from colloidal gelatin was assessed by visualizing Cy5-labelled eGFP mRNA incorporated within the NPs within the microfluidic chip (Figure 2A). There were substantial differences between the autofluorescent properties of the two types of gelatin, where negatively charged Gel B exhibited more autofluorescence than its positively charged counterpart (Gel A). Nevertheless, individual MC3T3 cells with internalized fluorescence could be identified through microscopy (Figure 2B and Figure S3). Two hours after transfection, abundant uptake of transfection complexes co-formulated with Gel A or Gel B was observed throughout the entire microfluidic chip, regardless of the transfection complex. Moreover, the differences in the distribution of intracellular Cy5 signal between PF14 and LMM (punctate versus homogenous cytosolic) were very reminiscent of those that we had observed in the absence of colloidal gelatin (Figure S3).

**Figure 3:**
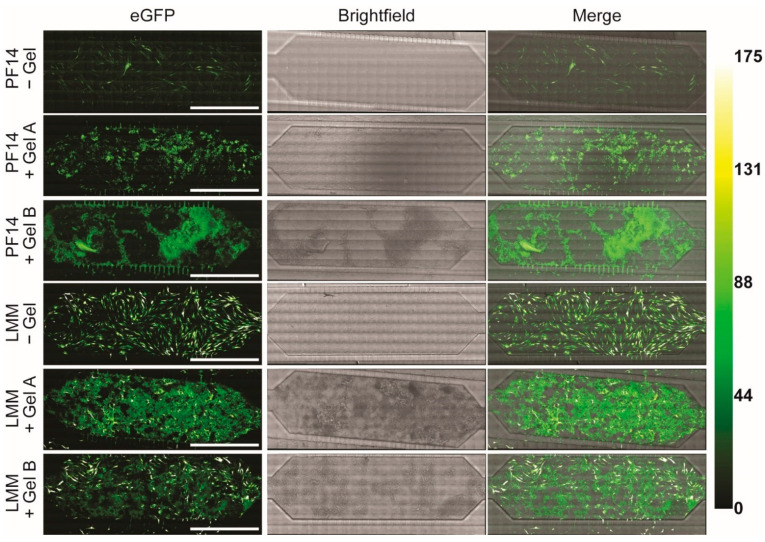
Expression of eGFP mRNA in the absence and presence of colloidal gelatin. Confocal microscopy images of eGFP expression in MC3T3 cells 24 h post-transfection. The conditions without gelatin were used to assess the impact of colloidal gelatin on transfection efficiency. The Green-Hot LUT on the right depicts the eGFP intensity. Brightness and contrast were individually adjusted for all images. Scale bars represent 1000 µm. eGFP, enhanced green fluorescent protein.

After validating mRNA uptake in the presence of colloidal gelatin NPs, the resulting eGFP expression was investigated. Importantly, we used an unlabeled eGFP mRNA for the expression analysis, which is expressed approximately five-fold more efficiently than its Cy5-labelled equivalent [20]. As expected, there were considerable differences in transfection efficiencies between the peptide-based PF14 and lipid-based LMM transfection complexes (Figure 3). We have previously demonstrated a 1–2 order of magnitude enhanced transfection efficiency of LMM compared to PF14 in 2D mRNA transfections [22]. Once again, Gel B produced more autofluorescence than Gel A (Figure S4). In the absence of colloidal gelatin, both LMM and PF14 showed abundant eGFP expression, although PF14 did so to a lesser extent. Unfortunately, in the presence of colloidal gelatin, the eGFP fluorescence was similar to autofluorescence for the PF14 conditions characterized by reduced eGFP expression. For LMM, reduced transfection efficiency was observed in the presence of colloidal gelatin. However, mRNA transfection of LMM co-formulated with Gel A or Gel B still showed widespread eGFP expression, which was well above autofluorescence and clearly represented the outlines of MC3T3 cells. Collectively, these results demonstrate the suitability of our microfluidic platform to simply and cost-effectively assess the influence of colloidal biomaterials on mRNA transfections.

## 4. Discussion

The development of novel therapies using biologics as drugs must consider physiological barriers encountered in vivo. Here, by means of by high-resolution imaging, we demonstrate the versatility of our microfluidic platform by showing the delivery of engineered proteins consisting of mCherry fused to an affibody targeting CAIX, as well as the testing of biomaterials for local mRNA delivery. 

**Figure 4:**
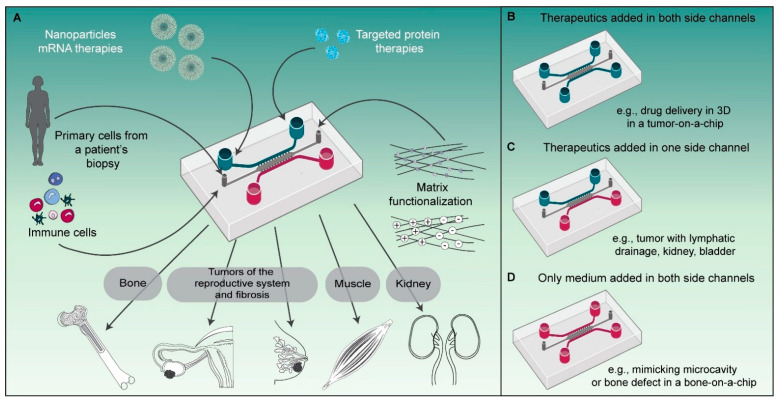
Schematic representation of the versatility of our microfluidic platform to study protein therapies and nanomedicines in various organ-on-a-chip systems. (**A**) Schematic with different parameters that can be included or modified in the microfluidic platform to mimic organ physiology and drug delivery. (**B**) Schematic of a microfluidic chip with drug delivery from both side channels as in the tumor-on-a-chip implementation utilized in this study to deliver anti-CAIX affibodies to tumor cells. (**C**) Alternative use of the microfluidic platform for mimicking the physiology of compartmentalized organ substructures from the kidney or bladder or the lymphatic drainage present in some tumors. (**D**) Schematic of the microfluidic chip with addition of only medium in the side channels, as studied in this paper, to mimic a bone-on-a-chip.

The application of microfluidic technology to mimic key tissue properties in organ-on-a-chip systems, particularly a fine control over the extracellular matrix properties, has rapidly grown over the last decade. The rapid accumulation of improvements in this technology, combined with an ever-increasing ease of use, is yielding improved methodologies to accurately predict the in vivo efficacy of investigational drugs [23]. From the perspective of investigating drug delivery processes, it is of particular importance that physical and (bio)chemical barriers are realistically mimicked, including the endothelial barrier, the matrix properties and densities, and the identity and density of the cells in the tissue (Figure 4). With respect to cell identity, incorporating cells with proper identities, e.g., from patient-derived materials or induced pluripotent stem cells (iPSCs), offers novel research possibilities. Control over environmental conditions that influence cell phenotypes, such as oxygen levels and the chemical composition of the microenvironment, is equally important because it greatly affects the pool of available cell surface receptors [12] and the behavior of cells in the microenvironment. Combined with the ability to visualize drug delivery processes using high-resolution fluorescence microscopy on-chip, microfluidic technology is poised to play an increasingly important role in drug delivery research.

The versatility of our microfluidic platform allows us to employ the same design to replicate different organ-level physiologies simply by altering how we prepare and apply the device (Figure 4). In this study we have shown targeted drug delivery in 3D using our platform as a tumor-on-a-chip system with delivery in two directions from the side (vessel-mimicking) channels towards the tumor compartment (Figure 4B). Using the same design, it would also be possible to mimic the physiological compartmentalization, e.g., by incorporating separating cell layers with an apical and basal orientation, as observed in some organs such as the kidney, or to mimic the (poor) lymphatic drainage observed in tumors, by perfusing medium or a therapeutic through only one of the side channels, creating a unidirectional flow (Figure 4C). The increased interstitial pressure that is associated with poor lymphatic drainage in vivo could also be replicated in this configuration if one of the side channels was partially blocked while the other one was perfused. In other cases where the perfusion of therapeutics is not required, like in our bone-on-a-chip model, the studied cells and materials can solely be added in the main compartment, while nutrient delivery to the cells is ensured through the side channels (Figure 4D).

In order to use such a versatile and multipurpose microfluidic chip platform, the initial investment costs are <USD 1000 for the epoxy resin SU-8 mold and about USD 5000 for a plasma cleaner to bond the PDMS devices to the glass coverslips. These investments are likely acceptable even for laboratories not specializing in organ-on-a-chip technology. The initial master mold has to be made in a cleanroom environment, which is available on many (medical) university campuses. Alternatively, such a mold can be acquired through a commercial party. Notably, the small volumes used in our microfluidic platform constitute an almost 10-fold reduction in materials needed for experiments, further rationalizing the initial investment costs. Besides the costs, another factor that needs to be taken into account is the training of researchers in the application of microfluidic technology. While the application of microfluidic technology is becoming more standard in many laboratories around the world, expertise is still required to address the day-to-day challenges, such as mastering the microfabrication process for obtaining leakproof devices, maintaining the sterility and viability of the cell cultures, as well as avoiding experiment-distorting factors such as bubbles or high pressures, e.g., during cell or matrix loading.

While several receptors have been targeted on-chip by us and others in previous work using protein-based agents [8,9,24,25], we report, for the first time, successful targeting of the well-established tumor target CAIX on-chip by CAIX-specific affibodies. For the validation of CAIX targeting, we employed renal carcinoma cell lines that overexpress CAIX, reflecting the CAIX induction upon VHL mutation as widely seen in renal carcinomas [13]. Since CAIX is also upregulated under hypoxic conditions, as a next step, it will be of interest to study CAIX targeting under hypoxic conditions, which can be achieved by employing microfluidic devices that control the oxygen tension on-chip [26,27]. To predict the potential side effect profile of CAIX-targeting agents in humans, it will be important to establish organ-on-a-chip systems that link tumor tissue with multiple tissues in an in vitro circulation, often referred to as so-called body-on-a-chip systems [28]. For such a system, a careful evaluation of the expression level of the targeted receptor in normal tissue is needed, which, in the case of CAIX, also involves expression in the gallbladder, pancreas, and stomach. Modules of these tissues will have to be incorporated in order to predict side effects that may be dose-limiting in vivo properly. Of note, CAR T-cells targeted to CAIX have shown liver toxicity due to CAIX expression in normal bile duct epithelium [29]. 

Furthermore, our study represents the first example of successful delivery and transfection of eGFP mRNA processed into lipid- or peptide-based transfection complexes from colloidal biomaterials. mRNA technology has gathered enormous attention over the last two years due to the success of mRNA vaccines in COVID-19 [30,31,32]. It is additionally being evaluated in many different disease applications, with clinical trials of therapeutic vaccines already being performed in cancer [33,34] and regenerative angiogenesis in areas such as diabetic ulcers [35]. Our results now offer the opportunity to study local delivery of mRNA from colloidal biomaterials to stimulate bone regeneration.

So far, these approaches have been limited to fibrous collagen matrices. Colloidal biomaterials, in turn, offer advantages for the delivery of mRNA NPs with respect to injectability into bone defects and self-healing capacity [36]. It is important to understand, however, to which degree these biomaterials are compatible with mRNA formulations. Here, the microfluidic system offers major advantages over classical formats, e.g., in microtiter plates. With a minimal volume of the cavity, relatively high densities of the material can be achieved with minimal sample consumption. 

In conclusion, our platform can be easily adapted to study drug delivery across different disease areas and with different targeted therapies. By controlling key parameters and high-resolution imaging, our platform represents an important additional technology in the field of drug delivery between more classical cell-culture systems and in vivo studies.

## Data Availability

The data presented in this study are available on request from the corresponding author.

## Supplementary Materials

The following are available online at https://www.mdpi.com/article/10.3390/pharmaceutics13111944/s1, Table S1. Quantitative results of DLS measurements. Averages and standard deviations reflect four repeated measure-ments. PdI: polydispersity index. Figure S1: Purity and activity of mCherry-affibody fusion proteins in 2D cell culture and morphology of tumor cells in different growth conditions. Figure S2: Characterization of peptide-based (PF14) and lipid-based (LMM) mRNA transfection complexes. Figure S3: Comparison of Cy5-eGFP NP uptake in the presence and absence of colloidal gelatin. Figure S4: Expression of eGFP mRNA in the absence and presence of colloidal gelatin. Video S1: SK-RC-52 cells incubated on-chip with anti-CAIX affibody. Video S2: SK-RC-17 cells incubated on-chip with anti-CAIX affibody. Video S3: SK-RC-52 cells incubated on-chip with ZTAQ affibody. Video S4: SK-RC-17 cells incubated on-chip with ZTAQ affibody.
