# Supplementary material for: Mimicking the Biology of Engineered Protein and mRNA Nanoparticle Delivery Using a Versatile Microfluidic Platform
Source: Pharmaceutics. 2021 Nov 17;13(11):1944. doi: 10.3390/pharmaceutics13111944 (PMC8624409; doi:10.3390/pharmaceutics13111944)
Supplement: Supplementary file 1 [file pharmaceutics-13-01944-s001.zip › pharmaceutics-1440890-supplementary.pdf]

pharmaceutics MDPI

Supplementary Materials: Mimicking the Biology of Engineered Protein and mRNA Nanoparticle Delivery Using a Versatile Microfluidic Platform

Valentina Palacio-Castañeda, Rik Oude Egberink, Arbaaz Sait, Lea Andrée, Benedetta Maria Sala, Negar Hassani Besheli, Egbert Oosterwijk, Johan Nilvebrant, Sander C. G. Leeuwenburgh, Roland Brock and Wouter P. R. Verdurmen

Table S1. Quantitative results of DLS measurements. Averages and standard deviations reflect four repeated measurements. PdI: polydispersity index.

Sample	Z-Average (Ø nm)	PdI	Attenuator	Intercept	Derived Counts
PF14 + eGFP	80.2 ± 1.9	0.269 ± 0.005	8	0.923 ± 0.001	10,384 ± 41
LMM + eGFP	525.8 ± 105.2	0.416 ± 0.072	8	0.915 ± 0.003	7448 ± 1,258

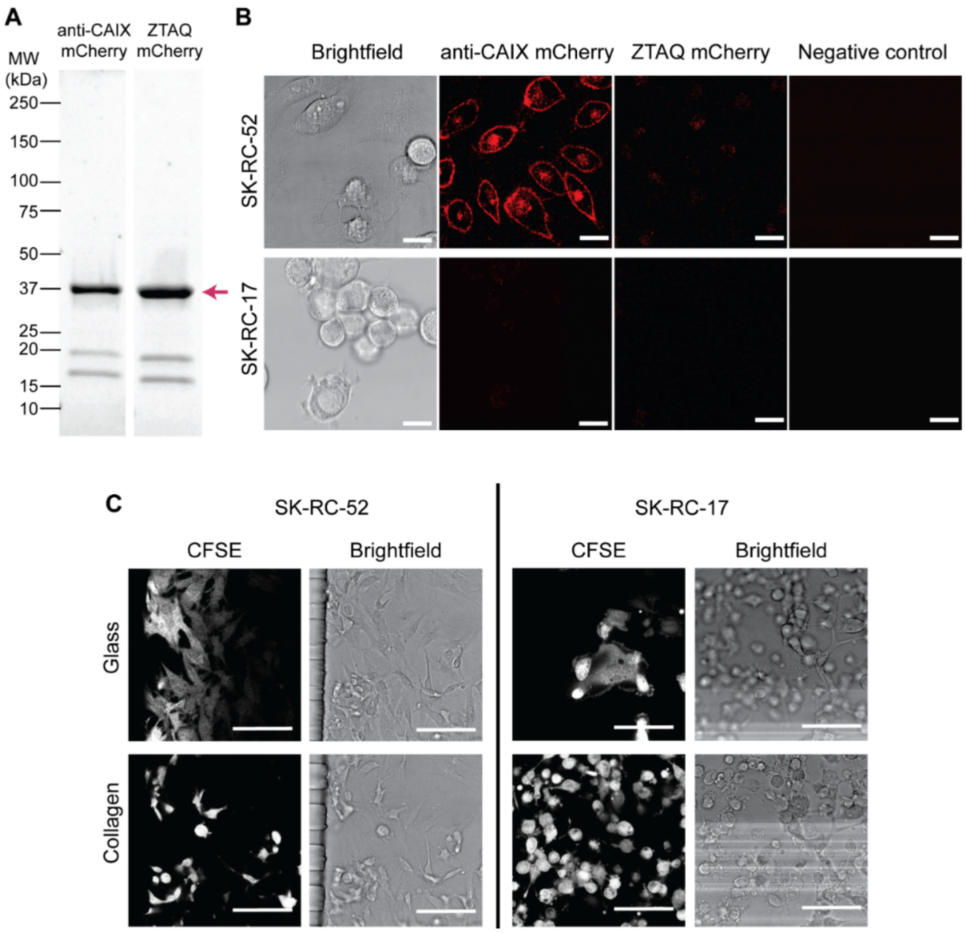

Figure S1. Purity and activity of mCherry-affibody fusion proteins in 2D cell culture and morphology of tumor cells in different growth conditions (**A**) Stain-free SDS-PAGE showing the purified mCherry fusions of CAIX-binding and non-targeting control ZTAQ affibodies, with a size of ~35 kDa. A minor degree of degradation is observed between 15-20 kDa. (**B**) Confocal microscopy images after a one-hour incubation with the anti-CAIX affibody-mCherry fusion on CAIX-positive SK-RC-52 or CAIX-negative SK-RC-17 cells in 2D, including controls. The brightfield images show the morphology of the cells in 2D cultures. Scale bars represent 20 µm. (C) Confocal images of CAIX-positive SK-RC-52 or CAIX-negative SK-RC-17 cells growing at different heights in the microfluidic device. Scale bars represent 100 µm. CFSE, carboxyfluorescein succinimidyl ester.

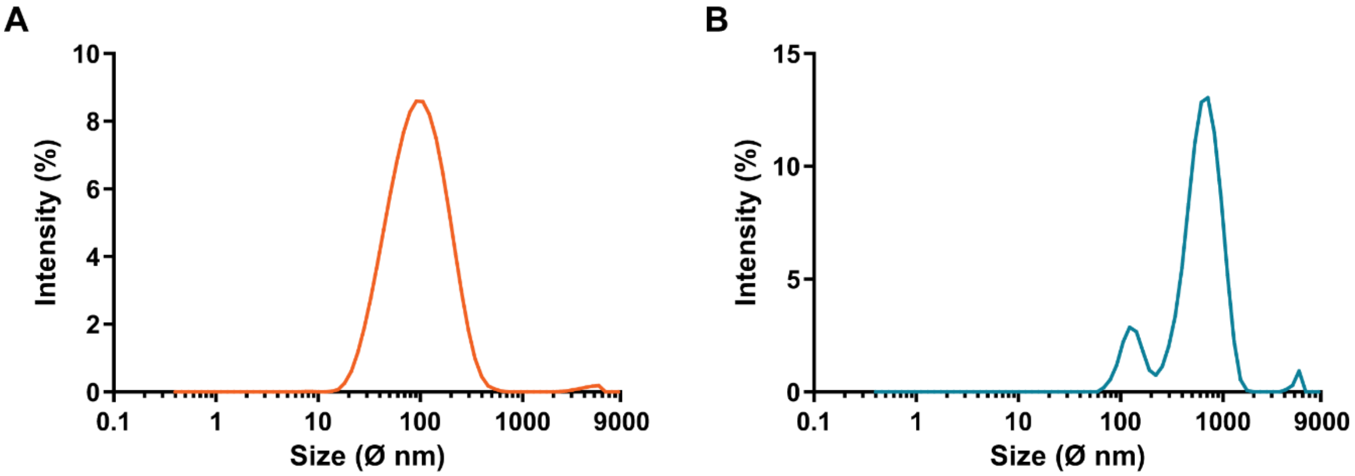

Figure S2. Characterization of peptide-based (PF14) and lipid-based (LMM) mRNA transfection complexes. (**A**) Intensity-based particle size distribution of PepFect14 formulated with eGFP mRNA. (**B**) Intensity-based particle size distribution of Lipofectamine MessengerMAX formulated with eGFP mRNA.

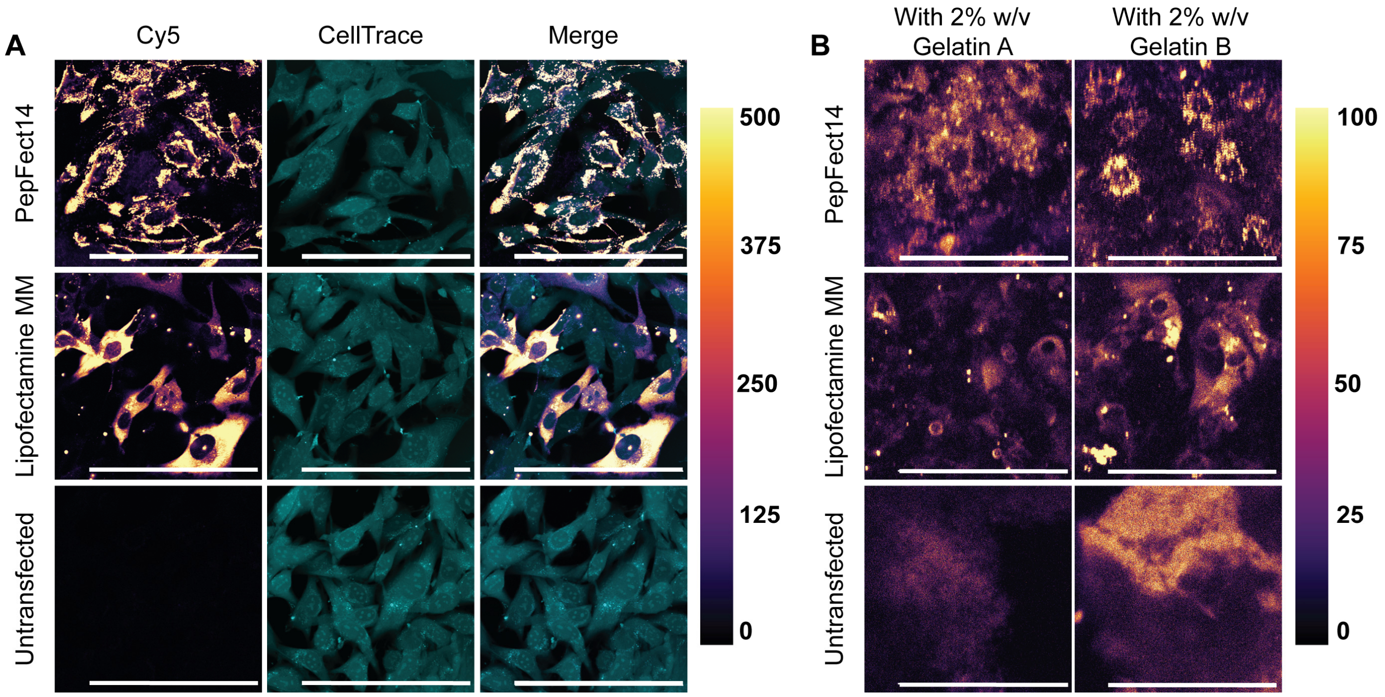

Figure S3. Comparison of Cy5-eGFP NP uptake in the presence and absence of colloidal gelatin. (**A**) Uptake of Cy5-eGFP mRNA transfection complexes by MC3T3 cells in Ibidi µ-slide 8 well chambered coverslips (stained with CellTrace Yellow) in the absence of colloidal gelatin. (**B**) Uptake of Cy5-eGFP mRNA transfection complexes by MC3T3 cells seeded in microfluidic chips in the presence of 2% w/v colloidal gelatin. The mpl-inferno LUT depicts the Cy5 intensity, and all Cy5 images were calibrated equally across conditions - per panel - according to the corresponding calibration bar on the right. Scale bars represent 150 µm. Cy5, Cyanine5.

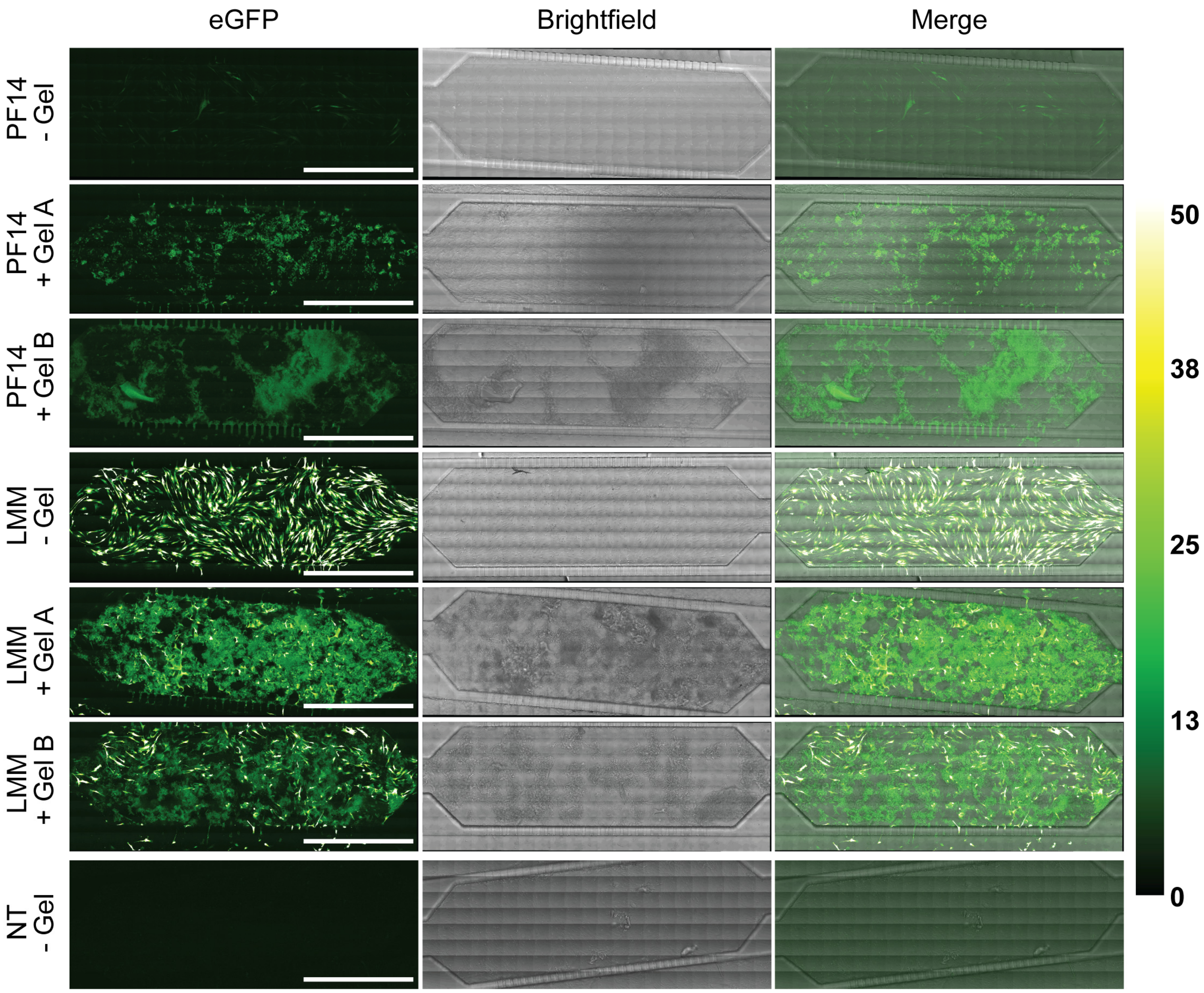

Figure S4: Expression of eGFP mRNA in the absence and presence of colloidal gelatin. Confocal microscopy images of MC3T3-associated expression, 24 hours post-transfection. The conditions without gelatin were used to assess the impact of colloidal gelatin on transfection efficiency. The Green-Hot LUT depicts the eGFP intensity, and the brightness and contrast were equally adjusted across conditions according to the corresponding calibration bar on the right. Scale bars represent 1000 μm eGFP, enhanced Green Fluorescent Protein; NT, non-treated.
